# Five-year Follow-up and Clinical Outcome in Euthyroid Patients with Thyroid Nodules

**DOI:** 10.2478/raon-2021-0025

**Published:** 2021-05-31

**Authors:** Katica Bajuk Studen, Simona Gaberscek, Edvard Pirnat, Katja Zaletel

**Affiliations:** 1 Department of Nuclear Medicine, University Medical Centre Ljubljana, LjubljanaSlovenia; 2 Faculty of Medicine, University of Ljubljana, LjubljanaSlovenia

**Keywords:** thyroid, nodule, goiter

## Abstract

**Background:**

Thyroid nodule diagnosis has become increasingly frequent. Defining optimum surveillance intervals for patients with unsuspicious thyroid nodules remains a challenge. This was a single centre cohort study in which patients diagnosed with unsuspicious thyroid nodules in whom no treatment was indicated were invited for re-evaluation 5 years after the diagnosis. The primary end point of the study was to estimate the change in nodule size with thyroid ultrasound (US) and the secondary end point was to assess the need for clinical management 5 years after the diagnosis.

**Patients and methods:**

Baseline patient parameters and ultrasound characteristics of the nodules were retrospectively collected. At follow-up, thyroid ultrasound was performed.

**Results:**

A hundred and eighteen (107 women / 11 men, aged 56.8 ± 13.4 years) patients were included in the study having 203 nodules at baseline, with mean largest nodule diameter 10.5 ± 7.4 mm. After 5 years, 58 (28.6%) nodules significantly increased in size, 27 (13.3%) decreased, and for 104 (51.2%) of nodules, no change in size was noted. Fourteen (6.9%) nodules disappeared. Additional 26 new nodules (mean largest diameter 7.7 ± 5.0 mm) in 16 patients were identified at follow-up. Regarding the clinical outcome, no new thyroid cancers were found. For 107 (90.7%) patients no further management was indicated. Five (4.2%) patients were referred to thyroidectomy because of the growth of the nodules. Two (1.7%) patients were treated for hyperthyroidism. Four (3.4%) patients did not complete the study.

**Conclusions:**

We report a single centre experience of the natural history of unsuspicious thyroid nodules. Our results showed that 71.4% of such nodules remained stable in size, decreased or even disappeared and that the vast majority of the patients remained clinically stable with no need for treatment 5 years after the diagnosis.

## Introduction

Thyroid nodules are discrete lesions within the thyroid gland that are morphologically distinct from the surrounding thyroid parenchyma.^1^ Currently, thyroid nodule diagnosis has become increasingly frequent due to incidental findings in different imaging tests performed for reasons unrelated to thyroid pathology.^2^, ^3^ The prevalence of thyroid nodules detected by thyroid ultrasound (US) in unselected populations was reported to be of up to 50% in adult females and 30% in adult males.^4^

The initial evaluation of patients with thyroid nodules consists of careful clinical, imaging and laboratory assessment, often aided by US-guided fine needle aspiration biopsy (FNAB). It should identify a small subgroup of nodules that either harbour thyroid cancer (approx. 10%), cause compressive symptoms (approx. 5%) or progress to functional disease (approx. 5%) and therefore need further clinical management.^1^,^5^,^6^ The rest of the nodules can safely be managed with a surveillance program. However, since the knowledge of the natural history of thyroid nodules is incomplete, defining optimum surveillance intervals still remains a challenge with goals not to miss out a clinically significant change of the nodule and not to overburden medical facilities and patients with unnecessary follow-up examinations. At present, long-term follow-up recommendations are mainly based on expert opinion consensus since there is no reliable method to identify patients likely to experience clinically significant nodule growth or change to malignancy.^1^,^6^

The purpose of our study was to establish a natural history of thyroid nodules in a cohort of euthyroid patients diagnosed with unsuspicious thyroid nodules. The primary end point of the study was to estimate the change in nodule size with thyroid US and the secondary end point was to assess the need for clinical management five years after the diagnosis.

## Patients and methods

The study was carried out in 2015–2017 as a 5-year follow-up of patients diagnosed with thyroid nodules at the Outpatient Thyroid Department of the University Medical Centre Ljubljana in the years 2010–2012. Only patients with unsuspicious nodules in whom at the time of diagnosis no treatment was indicated and who did not have autoimmune thyroid disease were included in the study. None of the patients was receiving levothyroxine therapy. Five years after the initial diagnosis, patients were invited by mail for clinical and US reevaluation. The study was performed in an iodine sufficient area.^7^ It was approved by the Republic of Slovenia National Medical Ethics Committee (No. 0120-721/2015-2). A written informed consent was obtained by all patients included in the study.

At baseline, a complete thyroid gland examination was performed, including clinical examination, thyroid US and measurement of thyrotropin (TSH), free thyroxine (fT_4_), free triiodothyronine (fT_3_), thyroid peroxidase antibodies (TPOAb), thyroglobulin antibodies (TgAb) and thyroglobulin (Tg). If the largest diameter of thyroid nodules exceeded 1 cm, thyroid scintigraphy was performed. To rule out malignancy, US-guided FNAB was performed in hypofunctioning thyroid nodules with suspicious US features.

At follow-up, the evaluation included clinical examination and thyroid US. If a significant increase of one or more thyroid nodules was confirmed or new nodules larger than 5 mm were detected, the patient was advised to proceed to a further complete thyroid gland examination that was scheduled at a separate visit. The complete thyroid gland examination included clinical examination, thyroid US, TSH and Tg measurement, FNAB for nodules with suspicious ultrasound appearance, and re-evaluation of the need for treatment.

All laboratory measurements were performed at the biochemical laboratory of the Department of Nuclear Medicine of the University Medical Centre Ljubljana. Serum concentration of TSH, fT_4_, fT_3_, TPOAb and TgAb was measured by ADVIA Centaur System (Siemens Medical Solutions Diagnostics). Reference values for TSH were 0.35– 5.5 mIU/L, for fT_4_ 11.5–22.7 pmol/L, for fT_3_ 3.5–6.5 pmol/L, and for TPOAb and TgAb less than 60 kIU/L. Thyroglobulin was measured by Kryptor platform (Brahms), based on TRACETM (time-resolved amplified cryptate emission) method with reference values between 0.5–58 μg/L.

Thyroid US was performed by 1 of 2 experienced thyroid specialists using an US machine (SSD-4000; Aloka Co, Ltd, Tokyo, Japan) with a 7.5-MHz linear transducer. The number of the nodules was recorded as well as their size in three dimensions. Multinodularity was defined as having more than 1 nodule. The volume of the nodules was calculated by the formula width x length x thickness x π/6. Suspicious ultrasound features were defined as at least one of the following: hypoechogenicity, irregular margins, taller-than-wide shape, and microcalcifications. A significant change in the size of thyroid nodule was defined as the increase or decrease that involved at least 2 nodule dimensions, each amounting to at least 2 mm and representing at least 20% of the baseline diameter.^1^,^9^ Thyroid autoimmunity was defined as hypoechoic US pattern and/or increased level of TPOAb and/or TgAb.

Thyroid scintigraphy was performed using a gamma camera equipped with a pinhole collimator (Siemens BASICAM) after intravenous administration of 100 MBq of Tc-99m pertechnetate.

Cytology results of FNAB were reported using the Bethesda system.^8^ Only patients with unsuspicious cytology results were included in the study (Bethesda category 2 as well as cysts, categorized as Bethesda 1).

The statistical analysis was performed with IBM SPSS Statistics Version 25 Software. Values are expressed as mean ± standard deviation (SD). For categorical baseline characteristics, differences between subgroups of patients were assessed by Pearson chi-square test. For continuous baseline characteristics, correlations with subgroup classification were assessed using Spearman’s rho test. Correlations of growth indicative variables with baseline parameters were calculated by using the Pearson correlation test (Pearson correlation coefficient, r). p-value below 0.05 was considered statistically significant.

## Results

**Figure 1:**
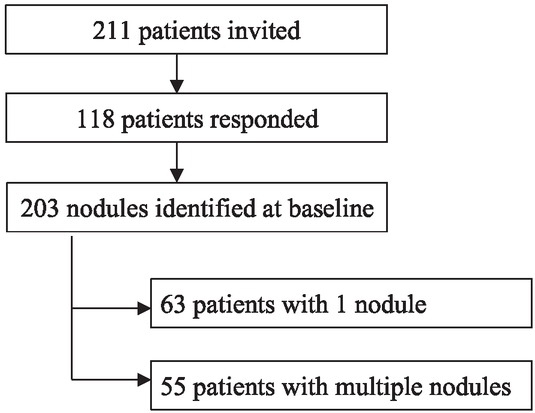
Flowchart explaining the recruitment process and the number of included patients and thyroid nodules.

The recruitment process is summarized in Figure 1. One hundred and eighteen patients were included in the study, with 203 thyroid nodules identified at baseline. One hundred and seven (90.7%) of the included patients were females and 11 (9.3%) were males. Basic characteristics of the included patients and the two subgroups with or without nodule growth are depicted in Table 1.

**Table 1:** Basic characteristics of patients included in the study (N = 118)

Parameter	All patients (N = 118)	Nodule Growth			New nodules detected		

		Without (N = 72)	With (N = 46)	p	Without (N = 102)	With (N = 16)	p
Age (years)	51.6 ± 13.4	51.8 ± 13.6	51.2 ± 13.2	0.81	51.2 ± 13.6	53.9 ± 11.9	0.46
TSH (mIU/L)	1.64 ± 0.85	1.73 ± 0.83	1.49 ± 0.85	0.07	1.63 ± 0.83	1.72 ± 1.00	0.80
Tg (μg/L)	42.4 ± 184.9	51.3 ± 237.2	28.8 ± 33.4	0.08	43.7 ±196.6	33.2 ± 70.5	0.68
Maximum diameter of the largest nodule (mm)	12.2 ± 8.2	11.7 ± 8.4	12.9 ± 7.7	0.11	12.2 ± 8.3	12.2 ± 7.6	0.86
Volume of the largest nodule (mL)	1.4 ± 2.4	1.4 ± 2.6	1.4 ± 2.1	0.17	1.4 ± 2.5	1.2 ± 1.4	0.70
Multinodularity (%)	55 (46.6%)	27 (37.5%)	28 (60.9%)	**0.02**	48 (47.1%)	7 (43.8%)	1.0

**Figure 2:**
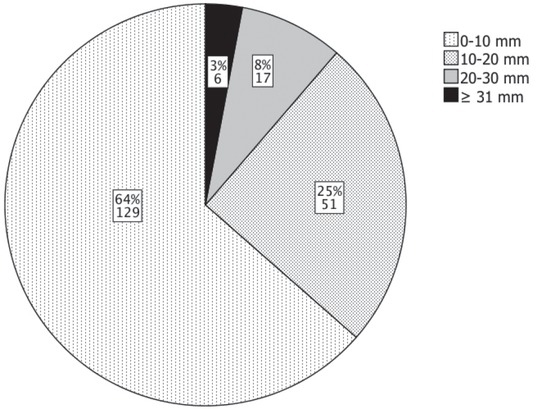
Nodule largest diameter distribution at baseline (N = 203).

Tg = thyroglobulin; TSH = thyrotropin;

Mean baseline largest diameter of the nodules was 10.5 ± 7.4 mm and mean baseline volume 1.1 ± 2.2 mL, with nodule baseline largest diameter distribution shown in Figure 2. In 55 nodules, FNAB was performed. The result of FNAB was Bethesda category 2 for 38 nodules and Bethesda category 1 (cyst) for 17 nodules.

**Figure 3:**
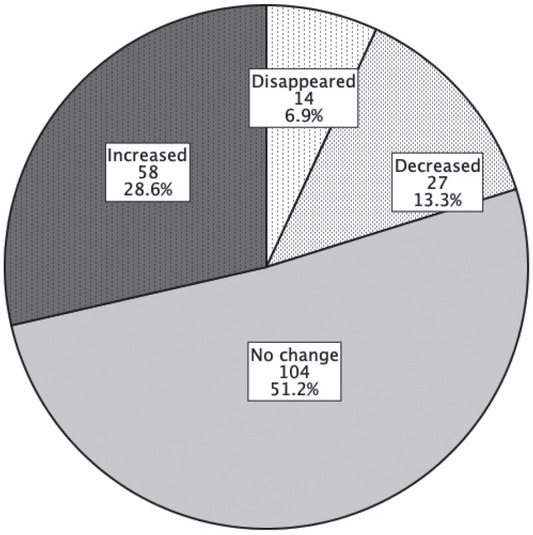
Growth status of the nodules after 5 years (N = 203). A significant change in the size of a thyroid nodule was defined as the increase or decrease that involved at least 2 nodule dimensions, each amounting to at least 2 mm and representing at least 20% of the baseline diameter.

After 5 years, 58 nodules significantly increased in size, 27 nodules significantly decreased in size; whereas for 104 of nodules, no significant change in size was noted (Figure 3). Furthermore, 14 of them disappeared. Twenty-six new nodules (mean largest diameter 7.7 ± 5.0 mm, mean volume 0.4 ± 0.8 mL) in 16 patients were found. The presence of multiple nodules was found to be significantly associated with nodule growth (Table 1).

**Figure 4:**
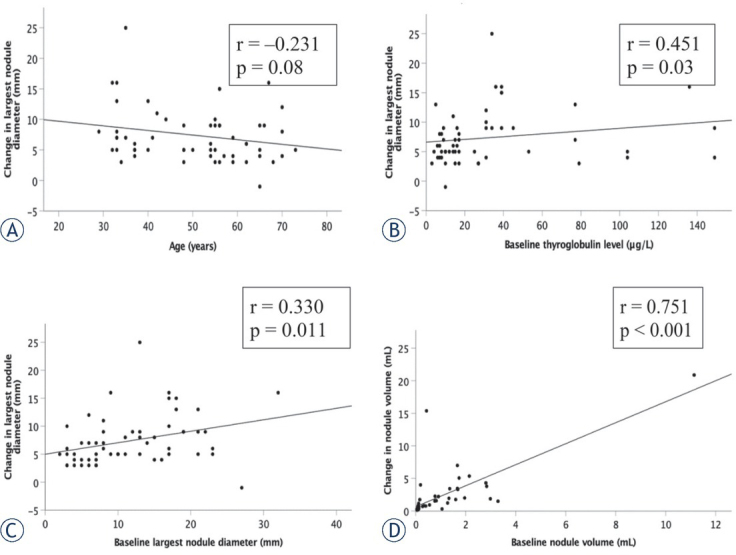
Correlations of baseline parameters with change in the largest nodule diameter and change in the nodule volume in nodules that significantly increased in size at the five-year follow-up (N = 58).

The parameters of 58 nodules that significantly increased in size were further analyzed. Correlations between baseline parameters (baseline age, baseline Tg, baseline largest nodule diameter and baseline nodule volume) and changes in the largest nodule diameter and nodule volume are depicted in Figure 4. Baseline TSH level was not found to correlate with changes in the largest nodule diameter nor nodule volume, r = – 0.119, p = 0.37 and r = – 0.082, p= 0.60, respectively (not shown in Figure 4). Baseline Tg level, baseline largest nodule diameter as well as baseline nodule volume positively and significantly correlated with nodule growth (p=0.03, p=0.011 and p<0.001, respectively).

### Clinical outcome after five years

No new thyroid cancers were found. For 107 (90.7%) patients no further management was indicated. Five (4.2%) patients were referred to thyroidectomy because of the growth of the nodules. Two (1.7%) patients were treated for hyperthyroidism (one received radioiodine treatment because of toxic multinodular goiter, another was treated with anti-thyroid drugs because of newly occurred Graves’ disease). Four (3.4%) included patients who were advised to proceed to a complete thyroid gland examination did not decide to do so for unknown reasons and were lost to follow-up.

## Discussion

In our study, we report a single center experience of the natural history of unsuspicious thyroid nodules in euthyroid patients for whom no treatment was indicated at the time of diagnosis. Our results show that 71.4% of such nodules remain stable in size, decrease or even disappear and that the vast majority of the patients remain clinically stable with no treatment indication five years after the diagnosis. The presence of multiple nodules in patients is associated with nodule growth. For nodules that grow, nodule’s growth positively correlates with the baseline Tg level, baseline largest nodule diameter as well as with the baseline nodule volume.

Previous studies have reported conflicting results regarding the natural course of thyroid nodules.^10^,^11^,^12^ These results could be due to the methodological problems – different, often short follow-up intervals and different cut-offs of change in nodule size were used, which are not easily reproducible. In our study, the size change of the nodule was considered significant if a change of 20% or more was recorded in at least 2 nodule diameters, with a minimum increase of 2 mm. This approximates a nodule volume change of 50% which represents the minimal significant and reproducible change in nodule size suggested to be applied in clinical investigations and practice.^1^,^6^,^9^ Our finding that after five years, most of the nodules remained stable, decreased or even disappeared is in agreement with a previously published study applying the same strict cut-off measure for a significant change in nodule size.^13^

Our finding that nodule growth was positively associated with the baseline largest nodule diameter and nodule volume as well as with the presence of multiple nodules is also in agreement with a previous report.^13^ As expected, no association of baseline TSH level with nodule growth was found in our study since only patients with TSH within normal limits were included.

The pathogenesis of thyroid nodules as well as their growth are influenced by genetic and environmental factors. Among environmental factors, iodine supply is probably the most important risk factor with nodular goiter being more prevalent in iodine deficient areas.^14^ Our study was conducted in an area that was iodine-sufficient for more than ten years before baseline evaluation of the patients.^7^ Therefore, the change in size of thyroid nodules reported in our study can be attributed to genetic and non-iodine related factors.^15^ However, firm data of relative contributions and causality of those factors is lacking and should be elucidated by future research.

Our finding that more than 90% of the patients five years after initial diagnosis did not need any further management of thyroid nodules supports our approach of a thorough first examination, which enables identifying a subgroup of patients who need treatment (due to malignancy, thyroid autonomy or compressive symptoms). It seems that such approach is more important than planning different follow-up strategies. Of note, patients with autoimmune thyroid disease with possible increasing TSH levels over time, patients with suspicious US features of thyroid nodules and those with inconclusive cytology reports were not included in the study. In such patients, follow-up is indicated.^1^,^6^

## Conclusions

In conclusion, our results support the approach that after a thorough first examination the majority of patients with unsuspicious thyroid nodules do not need frequent follow-up. Further research should elucidate the genetic determinants and biological characteristics of thyroid nodules that grow in time.

## References

[1] Haugen BR, Alexander EK, Bible KC, Doherty GM, Mandel SJ, Nikiforov YE (2016). 2015 American thyroid association management guidelines for adult patients with thyroid nodules and differentiated thyroid cancer: the American thyroid association guidelines task force on thyroid nodules and differentiated thyroid cancer. Thyroid.

[2] Russ G, Leboulleux S, Leenhardt L, Hegedüs L (2014). Thyroid incidentalomas: epidemiology, risk stratification with ultrasound and workup. Eur Thyroid J.

[3] Jamsek J, Zagar I, Gaberscek S, Grmek M (2015). Thyroid lesions incidentally detected by (18)F-FDG PET-CT – a two centre retrospective study. Radiol Oncol.

[4] Hegedüs L (2004). Clinical practice. The thyroid nodule. N Engl J Med.

[5] Russ G, Bonnema SJ, Erdogan MF, Durante C, Ngu R, Leenhardt L (2017). European thyroid association guidelines for ultrasound malignancy risk stratification of thyroid nodules in adults: the EU-TIRADS. Eur Thyroid J.

[6] Durante C, Grani G, Lamartina L, Filetti S, Mandel SJ, Cooper DS (2018). The diagnosis and management of thyroid nodules. JAMA.

[7] Zaletel K, Gaberšček S, Pirnat E, Krhin B, Hojker H (2011). Ten-year follow-up of thyroid epidemiology in Slovenia after increase in salt iodization. Croat Med J.

[8] Cibas ES, Ali SZ (2009). The Bethesda system for reporting thyroid cytopathology. Thyroid.

[9] Brauer VFH, Eder P, Miehle K, Wiesner TD, Hasenclever H, Paschke R (2005). Interobserver variation for ultrasound determination of thyroid nodule volumes. Thyroid.

[10] Quadbeck B, Pruellage J, Roggenbuck U, Hirche H, Janssen OE, Mann K (2002). Long-term follow-up of thyroid nodule growth. Exp Clin Endocrinol Diabetes.

[11] Alexander EK, Hurwitz S, Heering JP, Benson CB, Frates MC, Doubilet PM (2003). Natural history of benign solid and cystic thyroid nodules. Ann Intern Med.

[12] Erdogan MF, Gursoy A, Erdogan G (2006). Natural course of benign thyroid nodules in a moderately iodine-deficient area. Clin Endocrinol.

[13] Durante C, Costante G, Lucisano G, Bruno R, Meringolo D, Paciaroni A (2015). The natural history of benign thyroid nodules. JAMA.

[14] Carlé A, Krejbjerg A, Laurberg P (2014). Epidemiology of nodular goitre. Influence of iodine intake. *Best Pract Res Clin Endocrinol Metabol*.

[15] Knudsen N, Brix TH (2014). Genetic and non-iodine-related factors in the aetiology of nodular goitre. Best Pract Res Clin Endocrinol Metabol.

